# Genetic variation in *Mycobacterium tuberculosis* isolates from a London outbreak associated with isoniazid resistance

**DOI:** 10.1186/s12916-016-0659-6

**Published:** 2016-08-16

**Authors:** Giovanni Satta, Adam A. Witney, Robert J. Shorten, Magdalena Karlikowska, Marc Lipman, Timothy D. McHugh

**Affiliations:** 1 Department of Infection, Centre for Clinical Microbiology, University College London, London, UK; 2 Imperial College Healthcare NHS Trust, London, UK; 3 Institute of Infection and Immunity, St George’s, University of London, London, UK; 4 Public Health Laboratory Manchester, Manchester Royal Infirmary, Manchester, UK; 5 Royal Free London NHS Foundation Trust, London, UK; 6 UCL Respiratory, Division of Medicine, University College London, London, UK

**Keywords:** *Mycobacterium tuberculosis*, Whole-genome sequencing, Fitness, Mutation rate

## Abstract

**Background:**

The largest outbreak of isoniazid-resistant (INH-R) *Mycobacterium tuberculosis* in Western Europe is centred in North London, with over 400 cases diagnosed since 1995.

In the current study, we evaluated the genetic variation in a subset of clinical samples from the outbreak with the hypothesis that these isolates have unique biological characteristics that have served to prolong the outbreak.

**Methods:**

Fitness assays, mutation rate estimation, and whole-genome sequencing were performed to test for selective advantage and compensatory mutations.

**Results:**

This detailed analysis of the genetic variation of these INH-R samples suggests that this outbreak consists of successful, closely related, circulating strains with heterogeneous resistance profiles and little or no associated fitness cost or impact on their mutation rate.

**Conclusions:**

Specific deletions and SNPs could be a peculiar feature of these INH-R *M. tuberculosis* isolates, and could potentially explain their persistence over the years.

## Background

There has been a global increase in isoniazid (INH)-resistant (INH-R) tuberculosis (TB) [1]. This is important because INH resistance reduces the chance of successful TB treatment, can lead to the development and spread of multidrug-resistant (MDR) TB, and can reduce the effectiveness of INH preventive therapy [2]. The highest incidence of INH resistance is in Eastern Europe, where 44.9 % of new TB cases are INH-R. This staggering proportion compares to 13.9 % of all cases elsewhere [3].

The challenge of INH-R TB to public health is illustrated by an outbreak in London in which over 400 cases have been diagnosed since 1995 [4]. This is the largest reported outbreak in Western Europe. Conventional epidemiological analysis indicated that the patients in 50 % of cases were born in the UK, were of white or black-Caribbean ethnicity, and had a strong link to drug use and prison detention. Adherence to treatment was poor in one-third of patients and several went on to acquire further resistance, including MDR-TB [5]. A second clinically relevant feature of this outbreak is the high transmission of infection to contacts (11%) compared with other documented outbreaks (0.7–2%) [6]. This could not be explained by the epidemiological data, suggesting that other factors must be contributing to the extent of this outbreak.

As previously described [7], this outbreak does not follow the usual definition with a point source and serial transmission. In fact, it is constituted by multiple clusters (defined as samples related at ≥80 % similarity by IS6110 fingerprinting and starting from a minimum of two cases) over a period of several years. One of the biggest clusters is RFL15 (from Royal Free London, and initially called Lineage 15 by Dr Robert Shorten [8]). It is this cluster that forms the basis of our study. Demographic characteristics from this cluster are comparable to those from previous studies [9]. The proportion of male patients was 54.2 % and there was no difference between the ages of the patients in this study and those seen in the data of Maguire [9]. The majority of our patients with TB were black African (47.3 %), while 21.9 % were from South Asia.

It is worth acknowledging that RFL15 only represents a small sample of the ongoing outbreak but some of its features make it unusual. The cluster is characterized by persistent transmission over the years (from 2002 to 2007) and it is composed of a variety of strains with different antibiograms (including drug-susceptible, INH-, and streptomycin-monoresistant as well as MDR strains of *Mycobacterium tuberculosis*, MTB). Epidemiological factors may potentially explain some of the features: many patients in this outbreak were prisoners and drug users residing in the North London boroughs. Even if direct transmission could not be demonstrated, this localized prevalence does, however, indicate that strains are circulating within specific communities.

Several reports suggest that the acquisition of resistance leads to a reduction in the fitness of the affected strain, but the full picture is far from clear [10–12]. A spontaneous mutation that confers drug resistance should provide an advantage in an appropriately selective environment (i.e., patient on treatment). If the mutation affects an essential gene/function and causes a metabolic cost, then we could reasonably hypothesize that the mutant will be less “fit” than its sensitive precursor. However, this is not always the case, and the use of whole-genome sequencing (WGS) has confirmed the presence of compensatory mutations that maintain a high competitive fitness [13].

Clinical strains of MTB show a genomic diversity that varies from few single nucleotide polymorphisms (SNPs) [14] to large-scale genomic rearrangements [15]. The majority of deletions are considered to be present in genes encoding for proteins not essential for the pathogenesis of the disease, as in these analyses all strains were obtained from clinical cases with active TB. However, some deletions could conceivably result in a selective advantage at particular stages of infection or transmission, or even enable escape from the host immune response. Other deletions could confer a strong advantage, such as antibiotic resistance (an example of this being deletion of the *katG* gene, resulting in INH resistance [16]).

In this study we evaluated the genetic variation in a subset of clinical samples from the London INH-R TB outbreak with the hypothesis that these isolates have unique biological characteristics that have served to prolong the outbreak. A fitness assay, mutation rate estimation, and WGS were performed to test the hypothesis of selective advantage and compensatory mutations.

## Methods

### Selection of samples

As part of a previous project, MTB isolates from the Royal Free London NHS Foundation Trust were investigated between 2002 and 2007. A specific cluster (RFL15), defined as samples related at ≥80 % similarity (by IS6110 fingerprinting), was described as part of the London INH-R TB outbreak. All isolates available from that cluster were evaluated by fitness assay, for mutation rate, and by WGS. Clinical isolates were originally frozen at −80 °C and all experiments were performed directly from the original stock with only one passage. The reference strain MTB H37Rv (from Public Health England, National Collection of Type Cultures), one unrelated INH-R isolate, and two unrelated susceptible isolates were included as controls. Drug sensitivities were performed at the National Mycobacterium Reference Laboratory (Public Health England, London, UK) as part of the routine clinical service.

### Fitness assay and mutation rate

Fitness assays were performed as previously described [17]. Automated liquid culture in the MGIT system (MGIT960 Becton Dickinson, Oxford, UK) was used. Mutation rate estimation was performed on 7H10 agar plates (BD/Difco, NJ, USA) containing ciprofloxacin and using the p0 method as previously described [18].

### Extraction of DNA and whole-genome sequencing

Frozen stocks were cultured on Löwenstein-Jensen slopes and DNA was extracted as previously described [19]. WGS was performed using the Illumina HiSeq platform (Illumina, San Diego, CA, USA) at the Genomic Services and Development Unit, Public Health England, according to standard protocols. The required DNA concentration was between 10 and 30 ng/μl with a 260/280 ratio of at least 1.8.

### Bioinformatics analysis

Sequence data were aligned to the H37Rv reference genome (RefSeq: NC_000962.3) using BWA-MEM 0.7.12 [20] and sorted using SAMtools v0.1.19 [21]. All genome sites were called using SAMtools mpileup as described previously [22]. The variant sites were filtered based on the following criteria: mapping quality (MQ) of >30, site quality score (QUAL) of >30, ≥4 reads covering each site with ≥2 reads mapping to each strand, ≥75 % of reads supporting the site (DP4), and an allelic frequency (AF1) of 1. Phylogenetic reconstruction was performed using RAxML v8.2.3 [23] with a Generalised time reversible  (GTR) model of nucleotide substitution and a Gamma model of rate heterogeneity; branch support values were determined using 1000 bootstrap replicates. Branch SNP counts were estimated by ancestral sequence reconstruction performed with PAML v4 [24]. Circular plots were generated using Circos [25]. The Phylo-Resistance Search Engine (PhyResSE) [26] was used to determine lineages and clades. The full analysis pipeline can be downloaded and run from http://github.com/bugs-bioinf/satta-2016.

## Results

Sixteen clinical isolates and three unrelated control samples were originally available, but only 13 isolates and the controls (16 samples in total) were included in the genetic analysis due to DNA extraction failures. Resistance profiles are detailed in Table 1. All isolates were INH resistant, except 02:113 and 05:046, which were fully sensitive, and 02:302, 03:013, and 03:313, which were streptomycin monoresistant. Samples 04.018 and 07.116 had additional resistance. Despite the different sensitivity profiles, all isolates were included as part of RFL15 (Table 1) and for a wider comparative genetic analysis.

**Table 1:** List of *Mycobacterium tuberculosis* clinical strains from the selected cluster RFL15

Type	Isolate	Lineage (clade)	Resistant to	Sensitive to	Polymorphism for INH	Relative fitness to H37Rv
Cluster RFL15	02.113	Euro American (Cameroon)	-	H,R,Z,E,S	None detected	0.96
	**02.292**	**Euro American (Cameroon)**	**H**	**R,Z,E,S**	***inhA C → T***	**0.88**
	02:302^a^	Euro American	S	H,R,Z,E	None detected	1.05
	03.013	Euro American (Cameroon)	S	H,R,Z,E	None detected	0.89
	**03.039**	**Euro American (Cameroon)**	**H**	**R,Z,E,S**	***inhA C → T***	**0.82**
	03.303^a^	Euro American	H	R,Z,E,S	*inhA C → T*	0.92
	03.313	Euro American (Cameroon)	S	H,R,Z,E	None detected	0.98
	**04.018**	**Euro American (Cameroon)**	**H, R, clari, ethi**	**Z,E,S**	***inhA C → T***	**0.75**
	04.194	Euro American (Uganda)	H	R,Z,E,S	*kat*G S315T	0.96
	04:198^a^	Euro American	H	R,Z,E,S	*inhA C → T*	0.98
	**04.211**	**Euro American (Cameroon)**	**H**	**R,Z,E,S**	***inhA C → T***	**0.93**
	**04.493**	**Euro American (Cameroon)**	**H**	**R,Z,E,S**	***inhA C → T***	**1.01**
	**04.503**	**Euro American (Cameroon)**	**H**	**R,Z,E,S**	***inhA C → T***	**0.94**
	05.046	Euro American (Cameroon)	-	H,R,Z,E,S	None detected	1.03
	**07.116**	**Euro American (Cameroon)**	**H, ethi**	**R,Z,E,S**	***inhA C → T***	**0.99**
	07:118	Euro American (Cameroon)	H	R,Z,E,S	*kat*G S315T	0.82
H-resistant control	05.177 (Control strain 1)	Euro American (Uganda)	H	R,Z,E,S	*inhA C → T*	0.92
H-sensitive control	05.094 (Control strain 2)	Euro American (Uganda)	-	H,R,Z,E,S	None detected	0.88
H-sensitive control	04.011 (Control strain 3)	East African Indian (Delhi/CAS)	-	H,R,Z,E,S	*kat*G R463L (but no resistance)	0.81
	**Mutation rate - outbreak strains**			**Mutation rate - control strains**		
	1.3 × 10^−8^ × cell division			1.3 × 10^−8^ × cell division		

^a^no whole-genome sequence available as unable to extract enough DNA

The first two digits in the isolate number indicate the year of isolation, for example, 02:113 was isolated in 2002. Sensitivities were based on phenotypical testing (abbreviations: *Clari* clarithromycin, *Ethi* ethionamide, *H* isoniazid, *R* rifampicin, *Z* pyrazinamide, *E* ethambutol, *S* streptomycin). Only the genetic mutations conferring isoniazid resistance are reported for simplicity. Strains in bold are closely related on further phylogenetic analysis (see phylogenetic tree)

### Fitness assay and mutation rate

The fitness and the mutation rate of the resistant isolates were not different from either the reference strain H37Rv, the other susceptible isolates in the cluster, or unrelated INH-susceptible and INH-resistant samples (Table 1).

### Genetic analysis

**Fig. 1:**
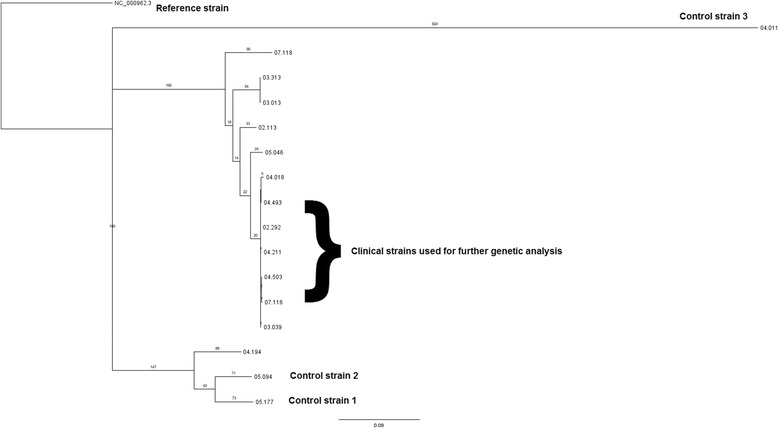
Phylogenetic reconstruction of the outbreak and control strains. All clinical outbreak strains, with the exception of 04.194, cluster together as part of RFL15. All outbreak isolates are of the Euro American (Cameroon) lineage, except sample 04.194, which is of the Euro American (Uganda) lineage. The control strains (05.177, 05:094, and 04.011) diverge as separate and independent strains: Euro American (Uganda) for the former two and East African Indian (Delhi) for the latter

Phylogenetic reconstruction (Fig. 1) showed that all the clinical isolates, with the exception of 04.194, cluster together as part of RFL15. In particular, outbreak samples 02.292, 03.039, 04.018, 04.211, 04.493, 04.503 and 07.116 appear to be closely related, despite being isolated over a period of 6 years. Samples 04.018 and 07.116 have also developed additional resistance. Other isolates, including 05.046, 02.113 (both drug sensitive), 03.013, and 03.313 (both streptomycin monoresistant only), diverge from the main group. The control samples (05.177, 05:094, and 04.011) are distinct as separate and independent strains.

#### Deletions

**Fig. 2:**
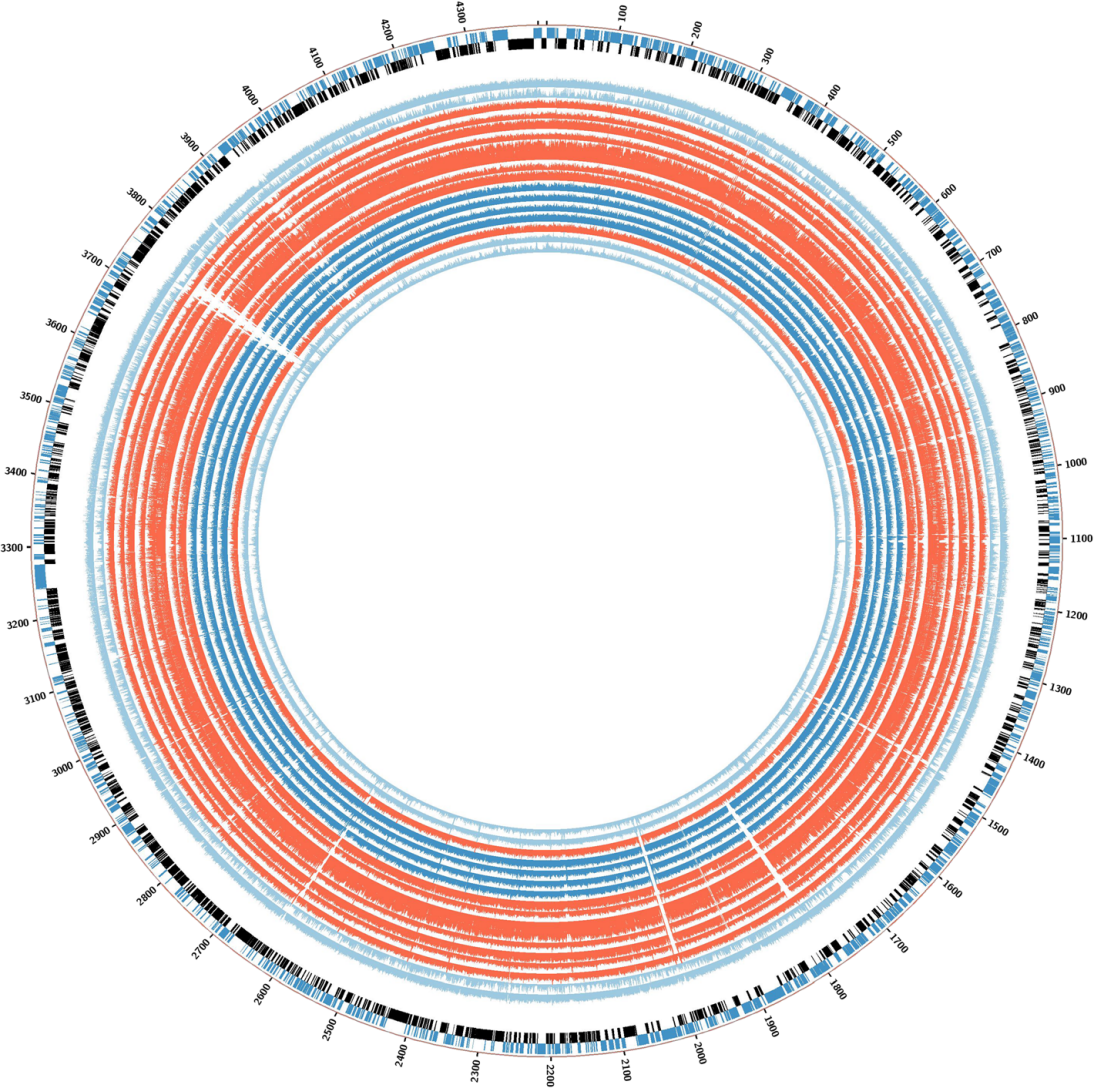
BLAST ring for the graphical representation of deleted genes in the isoniazid-resistant tuberculosis outbreak. Deleted regions are shown as *white empty spaces* in the alignment. The order is the same as for the phylogenetic tree. Strains 02.292, 03.039, 04.018, 04.211, 04.494, 04.503, and 07.116 (all closely related) are in *red*. Strains 05.046, 02.113, 03.013, and 03.313 are coloured *deep blue*, and control strains 05.177, 05.094, and 04.011 are *light blue* as *H37Rv*. Strain 04.194 is also shown in *red*

Based on the phylogenetic tree results, comparative analysis for the detection of deletions was initially performed between selected outbreak isolates (02.292, 03.039, 04.018, 04.211, 04.493, 04.503, and 07.116), the control strain 05.177 (with the same *inhA* mutation), and the outbreak strain 03.313 (streptomycin monoresistant). The selected isolates were originally chosen because they are closely related and so would prevent further genetic variation due to strain diversity. INH-R clinical samples demonstrated extensive deletions in 16 genes compared with the control strain used (05.177, still INH-R). Inclusion of sample 03.313 reduced the deleted gene set to 13 genes (the list of genes and their functional relevance is explained in Table 2; the BLAST ring is showed in Fig. 2).

**Table 2:** List of genes with complete deletion in the isoniazid-resistant outbreak strains

Genes name (function)	Isolates: 02.292, 03.039, 04.018, 04.211, 04.493, 04.503, 07.116	03.313	05.177 Control
*Phirv1 phage proteins* (part of genomic island)	✘	✘	✓
*Rv1673c* (conserved hypothetical protein)	✘	✘	✓
*Rv1675c* (probable cAMP and macrophage regulator)	✘	✘	✓
*plcD* (phosphoesterase)	✘	✘	✓
*Wag22* (antigen member, PE family)	✘	✘	✓
*Rv1760* (possible triacylglycerol synthase)	✘	✘	✓
*Rv1761c* (unknown protein)	✘	✘	✓
*Rv1762c* (unknown protein)	✘	✘	✓
*plcB* (phosphoesterase)	✘	✓	✓
*plcA* (membrane associated phospholipase)	✘	✓	✓
*PPE54* (part of PPE family)	✘	✓	✓
*PE_PGRS50* (antigen member, PE family)	✘	✘	✓
*PPE55* (part of PPE family)	✘	✘	✓
*Rv3349c* (probable transposase)	✘	✘	✓
*Rv3371* (possible triacylglycerol synthase)	✘	✘	✓
*Rv3486* (membrane protein, function unknown)	✘	✘	✓

Comparison is also made with possible outbreak strain 03.313 (phylogenetically related but only streptomycin monoresistant). The symbol ✘ indicates that the gene contains extensive deletions, while the symbol ✓indicates that the gene is still present. Deletions were found in 16 genes in the isoniazid-resistant tuberculosis outbreak isolates compared with the control strain 05.177. Additional comparison with another possible outbreak strain (03.313) reduced the total common deletions to 13 genes. PE and PPE: proline-glutamate (PE) and proline-proline-glutamate (PPE)

#### Single nucleotide polymorphisms

Comparative analysis was performed between the same selected outbreak isolates (02.292, 03.039, 04.018, 04.211, 04.493, 04.503, and 07.116) and the control sample 05.177 for the detection of SNPs. A total of 563 SNPs were identified. These were compared to a recent classification of MTB virulence factors [27], and 33 virulence genes were identified as affected by at least one SNP (Table 3).

**Table 3:** List of genes encoding for supposed virulence factors and affected by single nucleotide polymorphisms

Gene name	Function	Result	S/NS	Position	Amino acid change
*ctpA*	Cation transporting P-type ATPase	Reduced CFUs	NS	100895	A/S
*ctpI*	Cation transporting P-type ATPase	Reduced CFUs	S	130449	A/A
*mce1F*	Mce protein	Reduced tissue pathology and increased survival	NS	206453	G/A
*Rv0176*	Mce protein	Reduced CFUs, reduced tissue pathology, and increased survival	NS	208299	P/R
*pckA*	Iron-regulated phosphoenolpyruvate carboxykinase	Reduced CFUs	NS	252105	M/I
*pcaA*	Mycolic acid cyclopropane synthase	Reduced CFUs in lung	NS	561021	H/Y
*pstS1*	phosphate-binding lipoprotein component of inorganic phosphate transport system	Reduced multiplication	NS	1042991	P/A
*mprB*	Two component sensor kinase	Reduced CFUs in lung latent stage	S	1098698	G/G
*oppA*	Oligopeptide-binding lipoprotein component of peptide transport system	Reduced CFUs in organs in the chronic infection, increased survival	S	1433114	G/G
*oppD*	Oligopeptide-transport ATP binding protein	Reduced CFUs in organs in the chronic infection, increased survival	S	1434648	V/V
*irtA*	Iron-regulated transporter	Reduced CFUs in macrophages and lung	S	1514732	R/R
*irtB*	Iron-regulated transporter	Reduced CFUs in macrophages and lung	S	1515922	V/V
*Rv1410c*	Aminoglycoside/tetracycline-transport integral membrane protein	Reduced CFUs	S	1586360	A/A
*pks5*	Polyketide synthase	Reduced CFUs in organs	S	1726541	Y/Y
*eccB5*	EsX-5 type VII secretion system protein	Reduced CFUs	S	2017898	T/T
*eccD5*	EsX-5 type VII secretion system protein	Reduced CFUs	S	2032701	A/A
*mycP5*	Pro-rich membrane-anchored serine protease (mycosin)	Reduced CFUs	NS	2033748	G/A
*katG*	Catalase peroxidase peroxynitritase T	Reduced CFUs, resistance to INH	S	2155389	P/P
*Rv1931c*	Probable transcriptional regulatory protein	Reduced CFUs	S	2183054	G/G
*fadE18*	Acyl-CoA dehydrogenase	Reduced CFUs	S	2184781	G/G
*mce3A*	Mce protein, ? virulence factor	Reduced tissue pathology and increased survival	S	2210055	T/T
*dosT*	Histidine kinase response regulator	Moderate reduction CFUs	S	2273627	G/G
*pks12*	Polyketide synthase	Reduced CFUs	S	2294876	R/R
*pafA*	Proteasome accessory factor A	Reduced CFUs in organs and less tissue pathology	S	2355511	V/V
*mpa*	Proteasone ATPase	Reduced CFUs in organs and less tissue pathology	S	2375883	K/K
*Rv3083*	Probable monooxygenase	Reduced CFUs	NS	3448567	H/D
*nuoG*	NADH dehydrogenase	Increased animal survival and reduced CFUs in organs	S	3518089	G/G
*Rv3236c*	Probable conserved integral membrane transport protein	Reduced phagosome ROS production	S	3612571	V/V
*ponA2*	Penicillin-binding protein	Moderate reduction in CFUs	NS	4122882	R/S
*mmpL8*	Predicted drug exporter of the RND superfamily	Reduced CFUs in organs	S	4291134	R/R
*esxA*	Early secretory antigenic target	Reduced CFUs	S	4352875	N/N
*eccE2*	Type VII secretion system protein	Reduced CFUs	NS	4367659	V/A
*eccE2*	Type VII secretion system protein	Reduced CFUs	NS	4367911	I/T

Name of gene, function, and virulence result are shown, including if single nucleotide polymorphisms are synonymous or nonsynonymous, position, and amino acid change (if any) (adapted from [27])

*CFUs* colony-forming units, *NS* nonsynonymous, *ROS* reactive oxygen species, *S* synonymous

#### Insertions

Comparison between the outbreak isolate 04.211 and the reference strain H37Rv did not revealed the presence of any insertions (data not shown).

## Discussion

Previous epidemiological studies have described the evolution of INH-R strains to MDR strains via the development of resistance to rifampicin and other drugs. It was observed that only strains with the *KatG S315T* substitution were associated with successful transmission and the development of extra resistance [28, 29]. However, in the RFL15 cluster, *inhA C-T767* is the commonest mutation. In addition, the fitness and mutation rate of these resistant isolates is not affected. This indicates that if there were any fitness cost initially associated with the acquisition of resistance-conferring mutations, then it was either very small or the organisms have compensated for it since.

The application of WGS has allowed an in-depth genetic analysis of the selected outbreak isolates. At the phylogenetic level, it is interesting to note that strain 04.194 does not seem to belong to RFL15 as previously reported based on MIRU (Mycobacterial Interspersed Repetitive Units) and RFLP (Restriction Fragment Length Polymorphism) typing data, although it was previously considered an outlier and to be partially divergent because it carries the *katG* mutation instead of the *inhA* mutation (as sample 07.118). Also, at a deeper lineage analysis, it belongs to clade Uganda (still a European American lineage), while all other outbreak samples are clade Cameroon (Table 1 and Fig. 1). This supports the view that WGS offers a more precise means to delineate outbreaks [19].

The outbreak isolates show genetic variation with unique deletions and SNPs without additional insertions. Of the 13 genes identified as deleted, most of them are conserved hypothetical proteins and antigens whose functions are still unknown. These can be considered non-essential genes. Nevertheless, their deletion could potentially offer the advantage of escape from the host immune response and explain why these strains remain fixed in the community, prolonging the outbreak for years. In particular, some deletions are worth further attention and may confirm the hypothesis of escaping/reducing the host immune response:

- *Rv1675c* is a transcription factor known to be responsive to cAMP levels, and implicated in the biology of persistent TB infection [30]. It is a regulator of four different protein genes (*mdh*, *groEL2*, *Rv1265*, and *PE_PGRS6a*) during macrophage infection by MTB and they are likely to play a role in MTB-host interactions [31].
- the MTB genome contains four different *plc* genes (*plcA*, *plcB*, *plcC*, and *plcD*) that encode for the enzyme phospholipase C (PLC). This region frequently contains deletions [32]. The absence/altered function of these genes could in some way influence the overall PLC activity, resulting in an impaired ability to degrade the phagosome membrane and consequent persistence of the bacterium inside the macrophages. Alternatively, a reduction in the release of arachidonic acid could lead to a decreased influx of inflammatory cells to the first site of infection, thus allowing the MTB to partially escape an early immune response. It is probable that PLC is involved in a number of different mechanisms and is one part of a complex system that allows MTB to survive inside macrophages and sustain chronic infection [33–35].

It is difficult to interpret the real role of all 563 SNPs identified in this study. MTB genome contains 4 million base pairs and 3959 genes: 40 % of these have had their function characterized, while another 44 % have been proposed to have possible functional relevance. Musser et al*.* [36] studied 24 different genes encoding target proteins for the immune response of 16 different isolates of MTB: among these, 19 genes were unvaried and just six nucleotide polymorphism sites were identified in the five genes where variation occurred. They estimated an overall frequency of SNPs of about 1 per 10,000 bp (around 400 SNPs for the whole genome). Later, Fraser et al. [37] claimed a higher frequency of polymorphism (about 1 in 3000 bp) thanks to detailed comparative studies between H37Rv and CDC1551 strains. This study took into account both synonymous and nonsynonymous nucleotide polymorphisms, and it has to be considered that a precise evaluation of the frequency of SNPs is critical. Several other studies seem to confirm the value of one synonymous nucleotide change per 10,000 synonymous sites in structural genes [38–40]. Considering that the control sample 05.177 belongs to a different clade (Uganda) from the outbreak strains (Cameroon), the identified SNPs could reflect phylogenetic evolution rather than specific SNPs with functional relevance. Interestingly, mutations in 33 virulence genes were identified: their function is summarized in Table 3. However, only 10 of these 33 genes have a nonsynonymous SNP. Overall, these are reported to cause a reduction in the colony-forming unit and in phagosome production, and increase survival, thus allowing persistence in the human host. This further confirms the hypothesis that these isolates have unique biological characteristics that have served to prolong this outbreak, granting these strains the fascinating ability to persist in the host, potentially evading the immune response and allowing transmission to contacts (as confirmed by epidemiological data).

## Conclusions

Analysis of the genetic variations of INH-R TB clinical samples from the London outbreak suggests that this outbreak consists of successful, closely related, circulating strains with heterogeneous resistance profiles and mutations, and little or no associated fitness cost or impact on their mutation rate. Deletions and SNPs may be a peculiar feature of these isolates and can potentially explain the persistence of this lineage in the community and the prolongation of the outbreak for years. Further studies are needed to better understand the impact of these deleted genes in the pathogenesis of TB and if any of the virulence genes involved by SNPs can be used as drug targets for the developement of new compounds.

### Nucleotide sequence accession number

The sequence data have been deposited in the European Nucleotide Archive with the study accession number [PRJEB13764].
